# Effect of ferric citrate on hippocampal iron accumulation and widespread molecular alterations associated with cognitive disorder in an ovariectomized mice model

**DOI:** 10.1111/cns.70018

**Published:** 2024-09-09

**Authors:** Lingling Cui, Huijun Zhou, Yudan Hao, Xiaoli Yang, Zhiqian Li, Yuting Gao, Zhengya Zhang, Lina Ren, Linpu Ji, Ruijie Sun, Yibo Wang, Xian Wang

**Affiliations:** ^1^ College of Public Health Zhengzhou University Zhengzhou Henan China

**Keywords:** cognitive disorder, iron, ovariectomy, proteomics

## Abstract

**Objective:**

Nowadays, the prevalence of cognitive impairment in women has gradually increased, especially in postmenopausal women. There were few studies on the mechanistic effects of iron exposure on neurotoxicity in postmenopausal women. The aim of this study is to investigate the effect of iron accumulation on cognitive ability in ovariectomized mice and its possible mechanism and to provide a scientific basis for the prevention of cognitive dysfunction in postmenopausal women.

**Methods:**

Female C57BL/6N ovariectomized model mice were induced with ferric citrate (FAC). The mice were randomly divided into 5 groups: control, sham, ovariectomized (Ovx), Ovx + 50 mg/kg FAC (Ovx + l), and Ovx + 100 mg/kg FAC (Ovx + h). The impact of motor and cognitive function was verified by a series of behavioral tests. The levels of serum iron parameters, malondialdehyde, and superoxide dismutase were measured. The ultrastructure of mice hippocampal microglia was imaged by transmission electron microscopy. The differential expression of hippocampal proteins was analyzed by Tandem Mass Tag labeling.

**Results:**

Movement and cognitive function in Ovx + l/Ovx + h mice were significantly decreased compared to control and Sham mice. Then, iron exposure caused histopathological changes in the hippocampus of mice. In addition, proteomic analysis revealed that 29/27/41 proteins were differentially expressed in the hippocampus when compared by Ovx vs. Sham, Ovx + l vs. Ovx, as well as Ovx + h vs. Ovx + l groups, respectively. Moreover, transferrin receptor protein (TFR1) and divalent metal transporter 1 (DMT1) protein expression were significantly increased in the iron accumulation mice model with ovariectomy.

**Conclusion:**

Iron exposure could cause histopathological damage in the hippocampus of ovariectomised mice and, by altering hippocampal proteomics, particularly the expression of hippocampal iron metabolism‐related proteins, could further influence cognitive impairment in ovariectomized mice.

## Graphical abstract

Iron accumulation mice model with ovariectomy could lead to the decline of movement and cognitive function. Iron accumulation could cause histopathological damage in the hippocampus of ovariectomized mice and, by disturbing hippocampus proteome, particularly the expression of hippocampal iron metabolism‐related proteins, could further influence cognitive impairment in ovariectomized mice.

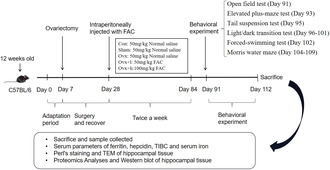

## 1 INTRODUCTION

Currently, more than 55 million people worldwide are living with dementia, with nearly 10 million new cases occurring each year.^1^ According to the report, the number of dementia patients is expected to reach 115.4 million, and the attributable dementia spending will reach $1.6 trillion (95% UI $0.9–$2.6) by 2050 worldwide,^2^ which negatively affect patients' health and place a substantial economic burden on individuals, families, and society. Cognitive disorder is an early symptom of dementia, which has attracted more and more attention.

It is now accepted that changes in age, genetics, and other factors can contribute to the onset and progression of the cognitive disorder.^3^ According to the latest review in 2023, among the 6.7 million elderly Alzheimer's dementia patients aged 65 years, 4.1 million were females and 2.6 million were males.^4^ A 16‐year prospective cohort study among older adults in China demonstrated that the prevalence of cognitive disorders in females and males was 27.5% and 15.7%, respectively.^5^ Brain imaging study found that compared with premenopausal women and men of the same age, perimenopausal, and postmenopausal women aged 40–60 years showed AD endophenotype.^6^ Therefore, more attention should be paid to cognitive status in postmenopausal women. It was worth noting that the iron content of the body was obvious elevated when the estrogen level was decreased after menopause. Evidence from French population among 3932 individuals showed that the mean level of serum ferritin in premenopausal and postmenopausal women was 32.6 and 64.2 μg/L, respectively. The risk of iron overload was higher in postmenopausal women than that in premenopausal women (8.9% vs. 2.8%).^7^ Previous studies found that middle‐aged and elderly people were more sensitive to iron overload and its resultant oxidative stress.^8^, ^9^ However, a lack of clear consensus remained on the impact of iron on the relationship between postmenopausal women and cognition‐related outcomes.

Iron, as an essential microelement in the physiology of human beings, is the component of multiple enzymes involved in a mass of metabolic processes. Evidence from a recent review showed that iron overload was closely related to various neurological diseases in middle‐aged and elderly people.^10^ Of note, iron is the most abundant metal in neurons, which holds a significant role in various biological pathways of the brain, such as synthesis of adenosine triphosphate and deoxyribonucleic acid (DNA), mitochondrial function as well as the cycling of neurotransmitters and myelin generation.^11^, ^12^ Brain iron abnormalities are associated with various neurodegenerative diseases,^13^ including Alzheimer's disease (AD), Huntington's disease (HD), Parkinson's disease (PD), etc. With increasing age, iron metabolism disorder could lead to iron accumulation and affect inflammatory processes, protein aggregation, and neuronal function.^13^ Excessive iron deposition was found in the substantia nigra and basal ganglia of PD patients at autopsy.^14^, ^15^ Besides, animal experiments also confirmed that iron accumulation could affect cognitive ability by behavioral experiments.^16^ Results from another animal study of 12‐month‐old C57BL/6 mice also showed that a low‐iron diet ameliorated sevoflurane‐induced cognitive deficits.^17^ Over the past years, many related researches made efforts to clarify the mechanisms underlying iron‐induced neurotoxicity, which remained unknown to date. Some previous research found that unstable iron could catalyze the production of reactive oxygen species (ROS) via the Fenton reaction and promote the oxidation of lipoxygenase and other peroxisomal oxidases.^18^ Meanwhile, mitochondrial damage caused by iron deposition could lead to oxidative damage to proteins, DNA, and lipids damaged.^19^, ^20^ In addition, disorders of iron metabolism could also lead to ferroptosis, which is an iron‐dependent form of programmed cell death.^21^

Thus, we propose that iron accumulation might contribute neurotoxicity in postmenopausal women through multiple pathways and eventually lead to cognitive dysfunction. To explore the effects of iron on cognition of postmenopausal women, an iron accumulation mice model with ovariectomy was established to mimic the postmenopausal women, and a series of behavioral tests, pathological, and biochemical analyses were conducted to identify its possible mechanism.

## 2 MATERIALS AND METHODS

### 2.1 Animals and ethical statement

**FIGURE 1:**
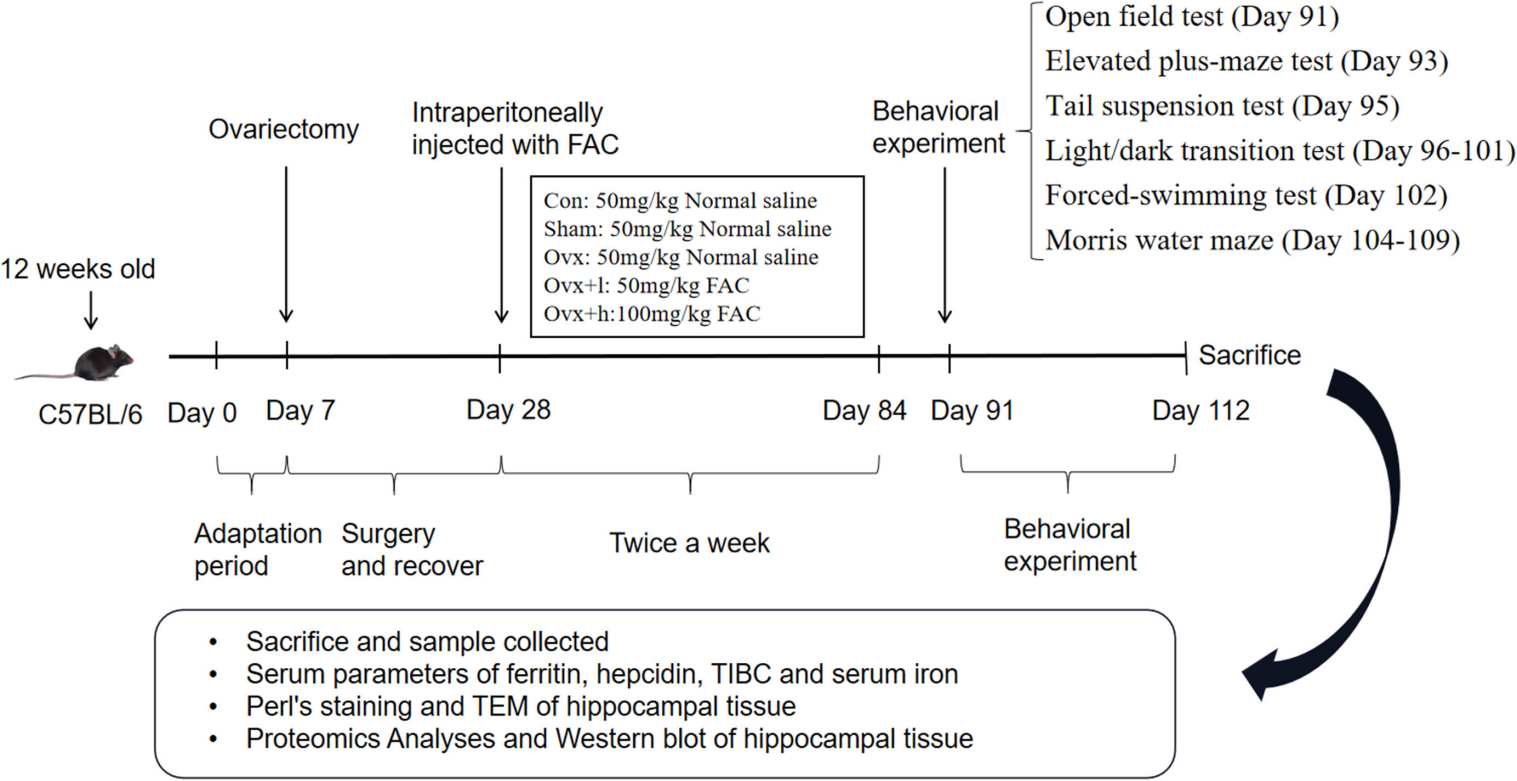
Experimental design. Chart illustrating our experimental design including surgery, drug administration, behavioral tests, Perl's staining, transmission electron microscopy (TEM), enzyme‐linked immunosorbent assay (ELISA), and protein expression. FAC, Ferric citrate.

All animal experiments followed guidelines and were approved by the Zhengzhou University Faculty of Life Science Ethics Committee (register number: ZZUIRB2021‐57). Female C57BL/6 mice obtained from Beijing Weitong Lihua Experimental Animal Technology Co. Ltd. were used in the present study. The animal license is SYXK (Henan) 2018‐0005. The mice were randomly divided into 5 groups, with 20 mice in each group including the control group (Con), sham‐operated group (Sham), ovariectomized group (Ovx), ovariectomized +50 mg/kg ferric citrate (FAC, sigma, America, 1185‐57‐5) group (Ovx + l), and ovariectomized +100 mg/kg FAC group (Ovx + h). Five mice died during the feeding process. After anesthesia, mice in the Ovx, Ovx + l, and Ovx + h were made to undergo bilateral ovariectomy, and only the adipose tissue around the ovaries was removed in Sham mice. The Con mice did not receive any intervention. Normal saline of 50 mg/kg was injected intraperitoneally in Con, Sham, and Ovx mice. The injections of normal saline or FAC were given twice a week for 8 weeks. The whole experimental procedure is shown in Figure 1.

Blood was collected by cardiac puncture and centrifuged to provide serum. Brain tissue was isolated, and hippocampal tissue was rapidly isolated on ice.

### 2.2 Serum estradiol (E_2_) assay

To verify whether the ovariectomy model was successful, serum was used for the determination of E_2_ levels using radioimmunoassay kits (Enzyme‐linked Biotechnology Co., Ltd. Shanghai, China, ml001962) according to the manufacturer's instructions.

### 2.3 Behavioral tests

#### 2.3.1 Open field test (OFT)

Anxiety‐like behavior was evaluated using the open field test (OFT), in which apparatus (50 × 50 × 50 cm) was placed in a quiet and dimly lit environment. The central area was defined as within a 25 × 25 cm range area near the middle. The mice were gently placed in the same corner of the testing chamber and were allowed 5 min of free movement, which was monitored by an automated video‐tracking system. Images of activity throughout 5 min were automatically analyzed using the SuperMaze (Shanghai XinRuan Information Technology Co., Ltd., Shanghai, China) Animal Behavior analysis program.

#### 2.3.2 Elevated plus maze test (EPM)

Anxiety‐like behavior was measured using the EPM (35 × 10 × 5 cm), which apparatus consisted of two opposite open arms, two opposite closed arms, and a central open platform (5 × 5 cm). Each mouse was placed on the central platform facing one of the open arms and freely explored the apparatus for 5 min. Parameters for each mouse were counted by the SuperMaze Animal Behavior analysis program.

#### 2.3.3 Tail suspension test (TST)

Depression‐like behavior was measured using the TST. The tail of the mouse was fixed on a hook with adhesive tape. Mice were placed approximately 30 cm above the ground, and a camera was placed in the horizontal direction with the mouse suspension device. Each mouse was suspended by its tail for 6 min. The first minute was the adaptation period, and the last 5 min was the official tail suspension time. The immobility time of the mice in the last 5 min was observed and recorded with a video camera.

#### 2.3.4 Forced‐swimming test (FST)

The FST was applied to assess rodent's depressive‐like behavior. Mice were placed into a clear Plexiglas cylinder (30 cm in height and 10 cm in diameter) filled up to two‐thirds with water (23–25°C) for a 6 min session. The first minute was the adaptation period, and the last 5 min was the official forced swimming time. The sessions were video‐recorded, and the duration of immobility, swimming, and struggling was measured.

#### 2.3.5 Morris water maze (MWM)

Cognitive function was evaluated using the MWM test, which was conducted in a circular tank (diameter, 150 cm; height, 50 cm) in a dimly lit room. A submerged escape platform (diameter, 5 cm) was equipped 1 cm below the milky water surface in one of the quadrants. Signs of black and white geometric figures were placed in the visible position of mice swimming in four directions of the pool. The MWM test training task, conducted over five consecutive days, entailed four trials daily from different orientations (60‐s cutoff). The trial was completed as soon as the mouse found the platform or more than 60 s. If the mouse could not find the submerged platform on a given trial, the mouse was guided to the submerged platform. The latency and path to the platform were tracked and recorded. On the sixth day, the platform was removed, and the mice were placed in the pool from the northeast corner. The residence time of the mice in the target quadrant where the platform was placed, the number of crossing platform in the quadrant and other relevant indicators within 1 min were recorded for further analysis.

#### 2.3.6 Light/dark transition test

Light–dark transition test was used to assess learning and memory ability. A 19.5 × 40 cm box consisting of 2 box compartments (light and dark) was used. The mice were placed in the light chamber, and the septum door between the light and dark chambers was opened after 60 s. Once the mice entered the dark chamber, the channel between the compartments was closed immediately, and the mice received electrical stimulation (50 Hz, 0.3 mA, 3 s). After the electrical stimulation, the mice were allowed to stay in the dark chamber for 10 s to end the training. The procedure was repeated after 2 min. If the mouse did not enter the dark box at 120 s, it was considered to have obtained passive avoidance. Each mouse received a maximum of three electrical stimuli. Tests were performed 1 h and 24 h after training to assess short‐term and long‐term memory. The dark chamber was not electrically stimulated during the test period, and the mice were placed in the bright chamber. After 20 s, the compartment door was opened, and the mice were free to explore for 300 s.

### 2.4 Histopathological assay

#### 2.4.1 Perls staining

The whole brain tissue in the fixative was removed and routinely dehydrated, embedded in paraffin. Coronal sections were made across the whole brain region, with a thickness of 3–5 μm. The detail of the procedure include: First, paraffin slices were dewaxed. After deparaffinization, the brain tissue sections were rinsed with distilled water for 1 min. The slices were immersed in Prussian blue staining solution for 20–30 min, and then the sections were removed and thoroughly rinsed with distilled water for 2–5 min. After that, nuclear fast red staining was performed. Finally, the slices were routinely dehydrated and transparent and sealed with neutral gum.

#### 2.4.2 Transmission electron microscopy (TEM)

After normal saline infusion, brain samples were rapidly immersed in glutaraldehyde (2.5%) and prefixed for 2–4 h. Tissues were rinsed 3 times with 0.1 mol/L phosphate buffer (PB, pH 7.4) for 15 min each time. The tissues were fixed in 1% osmic acid (resolved in 0.1 mol/L PB) for 2 h at room temperature and then rinsed 3 times with 0.1 mol/L for 15 min each time. The tissues were then dehydrated in acetone solutions at increasing concentrations and embedded in an epoxy resin. Then, the sections (70–90 nm) were stained with lead citrate and uranyl acetate. Ultrastructural images were then captured with a transmission electron microscope (Hitachi HT7700, Japan). All Perls staining and TEM slides were examined by two histopathologists who were blinded to the outcome of the mice.

### 2.5 Enzyme‐linked immunosorbent assay (ELISA)

In order to detect the levels of iron in serum, ferritin, hepcidin, total iron binding capacity (TIBC) (Elabscience Biotechnology Co., Ltd., China, E‐BC‐K071‐M), and serum iron (Solarbio Science & Technology Co., Ltd., Beijing, China, BC1735) were detected by ELISA according to the manufacturer's instructions.

### 2.6 Measurement of MDA and SOD levels

Malondialdehyde (MDA) and superoxide dismutase (SOD) are regarded as a significant biomarker of oxidative stress. The absorbance of MDA was recorded at 532 nm. SOD activity was measured at 420 nm. Hippocampal tissues from mice were treated with PBS homogenate and then centrifuged at 10,000 g for 15 min at 4°C. The MDA and SOD activity in Ipsilateral hippocampal tissue were measured following the manufacturer's instructions (Nanjing Jiancheng Bioengineering Institute, Nanjing, China, A003‐1 and A001‐3).

### 2.7 Proteomics analyses

#### 2.7.1 Tandem Mass Tag (TMT) labeling

Ipsilateral hippocampal tissues of three mice were selected from each group. Protein sample preparation and TMT labeling were performed according to the method described by Wang et al.^22^ Briefly, hippocampal tissues were ground to powder with liquid nitrogen, mixed evenly, and transferred to a centrifuge tube. Subsequently, appropriate amount of lysate was added to the centrifuge tube. Hippocampal tissue was disrupted by sonication on ice at 80 W for 2 min. The supernatant was collected and centrifuged at 12,000 × g for 10 min at 4°C. Protein concentrations were subsequently determined according to the instructions of the bicinchoninic acid assay (BCA, Leagene Biotechnology Co. Ltd., Beijing, China, PT0001) protein assay kit. Samples with 50 μg of protein were treated with dithiothreitol and further incubated with iodoacetamide. Digestion was carried out with 1:25 w/w w trypsin/Lys‐C overnight at 37°C and acidified with 1% formic acid (FA). Peptides were labeled with the TMT reagents at room temperature for 1 h. The reaction was terminated using 5% hydroxylamine. Labeled peptides of three mice from each group were mixed, desalted, dried, and dissolved in 100 μL 0.1% FA.

#### 2.7.2 Liquid chromatography–tandem mass spectrometry (LC–MS/MS) and bioinformatics analysis

Peptide analysis was conducted by LC–MS/MS according to a previous method.^23^ Briefly, samples were fed to Acclaim PepMap RSLC (75 μm × 50 cm) (RP‐C18, Thermo Fisher) column at a flow rate of 300 nL/min for separation. The primary MS mass resolution was set to 60,000, and the maximum injection time was 50 ms. The 20 highest peaks were scanned by MS/MS. All MS/MS map acquisition was done using high‐energy collision splitting in data‐dependent positive ion mode to characterize the properties of differential express proteins. In addition, the raw data of mass spectra were imported into Proteome Discoverer software (Version 2.4, Thermo Fisher Scientific, USA) for analysis of the spectra. Differentially expressed proteins were determined (requirements: adjusted *p* < 0.05 and fold change <1/1.2 or >1.2). The Gene Ontology database was used to analyze the biological process (BP), cellular component (CC), and molecular function (MF) of differentially expressed proteins according to their biological function and classification. Kyoto encyclopedia of genes and genomes (KEGG) database was used to analyze the main pathways involved in the differential proteins.

### 2.8 Western blot analysis

Proteins were obtained from the hippocampal tissue of mice. Tissues were weighed and homogenized in RIPA lysis buffer (Solarbio Science & Technology Co., Ltd., Beijing, China, R0010) containing 1 mM phenylmethanesulfonyl fluoride (PMSF; Solarbio Science & Technology Co., Ltd., Beijing, China, P0100). After the tissue was fully lysed, the supernatant was collected after centrifugation at 4°C for 5 min. The total protein concentration was quantified using the BCA. Protein samples were separated by electrophoresis of 7.5%–12.5% SDS‐polyacrylamide gel and transferred to polyvinylidene fluoride membranes (Millipore, Germany, ISEQ00010). The polyvinylidene fluoride membranes were blocked with 5% skim milk for 90 min on the shaker at room temperature, followed by overnight incubation with primary antibodies at 4°C and incubation with secondary antibodies at room temperature for 2 h. Signals were detected by chemiluminescence using ECL substrate (Biosharp, China, BL520A). Band density was quantified by NIH Image J software (Bethesda, MD, USA), and data were normalized to β‐actin. All of antibodies were obtained from Proteintech, China. The following primary antibodies were used: polyclonal rabbit anti‐transferrin receptor protein 1 (TFR1, 1:10,000, 66180‐1‐IG‐50); polyclonal rabbit anti‐divalent metal transporter 1 (DMT1, 1:2000, 20507‐1‐AP‐50), ponoclonal mouse anti‐ferroportin 1 (FPN1, 1:1000, 26601‐1‐AP‐50). The following secondary antibodies were used: HRP‐conjugated Affinipure Goat Anti‐Mouse IgG (1:10,000, SA00001‐1) and HRP‐conjugated Affinipure Goat Anti‐Rabbit IgG (1:10,000, SA00001‐2).

### 2.9 Statistical analysis

All quantitative data in this study were expressed as mean ± SEM. Statistical significance was considered for a two‐sided test with *p* < 0.05. Statistical analyses were carried out with SPSS (version 25.0, IBM Corp, Armonk, New York, USA). The data were tested with the Kolmogorov–Smirnov test to check the normality of the data. The difference between the groups was determined by one‐way analysis of variance followed by Tukey's post hoc test. When the normal distribution was not satisfied, Kruskal–Wallis rank sum test was used to compare the differences between groups. For each experiment, detailed statistical analysis and sample size were carefully reported in figure legends.

## 3 RESULTS

### 3.1 The ovariectomized mice model was successfully established

C57BL/6 female mice were subjected to bilateral ovariectomy, and then serum E_2_ levels were measured at 28th week when the mice were humanely sacrificed (Figure S1 and Table S1). E_2_ levels declined in Ovx groups accredited to removal of ovaries following bilateral ovariectomy. The result demonstrated that the removal of ovaries reduced the level of sex hormones in mice and successfully established the ovariectomized mice model.

### 3.2 Motor and cognitive defects were associated with iron accumulation in ovariectomized mice

**FIGURE 2:**
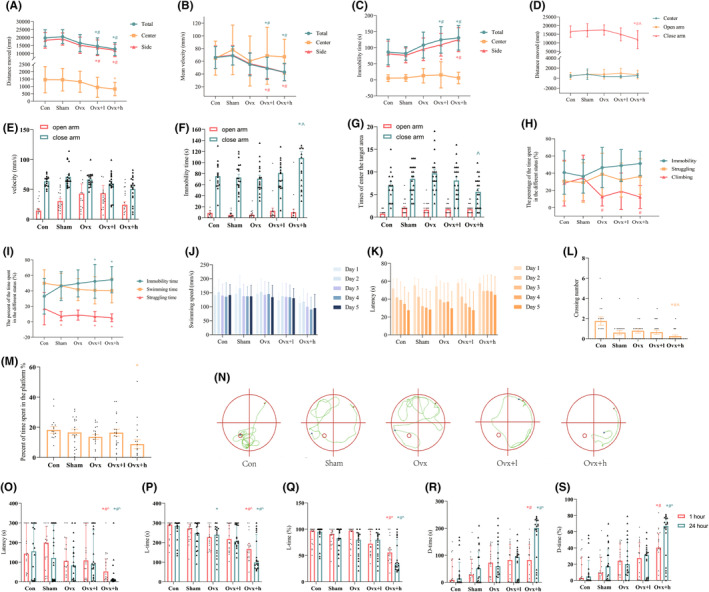
Iron‐induced cognitive disorder in ovariectomized mice. (A–C) The distance moved, mean velocity, and immobility time in total, center, and side areas, respectively, in the OFT. (D–G) The distance moved, mean velocity, immobility time, as well as the time of entering the target area in open and closed areas, respectively, in the EPM. (H) The percentage of immobility time, struggling time, and climbing time in the TST. Immobility was defined as when the mouse stopped struggling and the body remained vertically upside down. The struggle was considered to be a struggle movement that was visible in mice. Climbing was considered to be a powerful and vigorous movement of the mouse's limbs up and down. Immobility time (%) indicated the percentage of immobility time. Struggling time (%) indicated the percentage of struggling time. Climbing time (%) indicated the percentage of climbing time. (I) The percentage of immobility time, swimming time, and struggling time in the FST. (J, K) The swimming speed and latency of mice in the MWM training trials. (L, M) The crossing number and percent of time spent in the platform quadrant in the probe trial of the MWM. (N) Representative swimming trajectory of mice in the MWM. Red dots: Starting position; Blue dots: Ending position; Small red circles: The previous location of the platform. (O–S) The 1/24 h tested included latency, L‐time, L‐time%, D‐time and D‐time% in the light/dark transition test. The first time from the light chamber to the dark chamber was defined as the latency. Time spent in the light/dark chamber was defined as L‐time/D‐time. The percentage of time spent in the light/dark chamber was defined as L‐time%/D‐time%. Data were expressed as mean ± standard error of the mean (SEM). Data were analyzed using analysis of variance (ANOVA) with Tukey's post hoc test.When the normal distribution was not satisfied, Kruskal–Wallis rank sum test were used to compare the differences between groups Compared with Con mice, *p < 0.05. Compared with Sham mice, #*p* < 0.05. Compared with Ovx mice, ^*p* < 0.05.

A total of 95 mice were subjected to behavioral experiments. The results of OFT showed that, compared with Con and Sham groups, Ovx + l and Ovx + h mice had longer moving distance, slower mean velocity and the velocity, increased immobility time in total and side area (all *p* < 0.001, Figure 2A–C and Table S2). Data from EPM revealed that traveled distance and mean velocity of Ovx + h mice were significantly decreased compared to Con and Sham animals, the number of times in and out of different areas of Ovx + h mice were significantly decreased compared to Ovx animals (all *p* < 0.05; Figure 2D–G and Tables S3–S6). The results showed that the mice in Ovx + l and Ovx + h had anxiety‐like behavior. In addition, depression‐like behavior was observed in Ovx, Ovx + l, and Ovx + h mice assessed by TST and FST. The percent of climbing time in Ovx and Ovx + h mice were significantly decreased compared to Sham mice in TST; Similar to the TST, Ovx + l, and Ovx + h mice displayed significantly increased of the percent of immobility time and significantly decreased of the percent of struggling time with respect to Con mice in FST (all *p* < 0.05; Figure 2H,I and Tables S7 and S8).

Furthermore, a significant decline in memory consolidation was displayed in Ovx, Ovx + l and Ovx + h mice assessed by MWM, which results showed that, with the extension of training days, the time to reach the platform of mice in each group was shortened. Ovx + h mice underperformed given that they took less time to stay in the platform quadrant compared to Con mice (*p* < 0.05; Figure 2J–M and Tables S9–S11). The representative trajectory diagrams of each groups were shown in Figure 2N. Consistent with MWM finding, the same phenomenon was found in the Light/dark transition test. The latency, L‐time, and L‐time% of Ovx + l and Ovx + h mice in the 1 h and 24 h were shortened when comparing with the Con and Sham groups (all *p* < 0.05; Figure 2O–S and Tables S12–S15). All of the phenomena suggest differences in the groups in terms of motor and cognitive defects.

### 3.3 Iron accumulation affected the growth of mice

**FIGURE 3:**
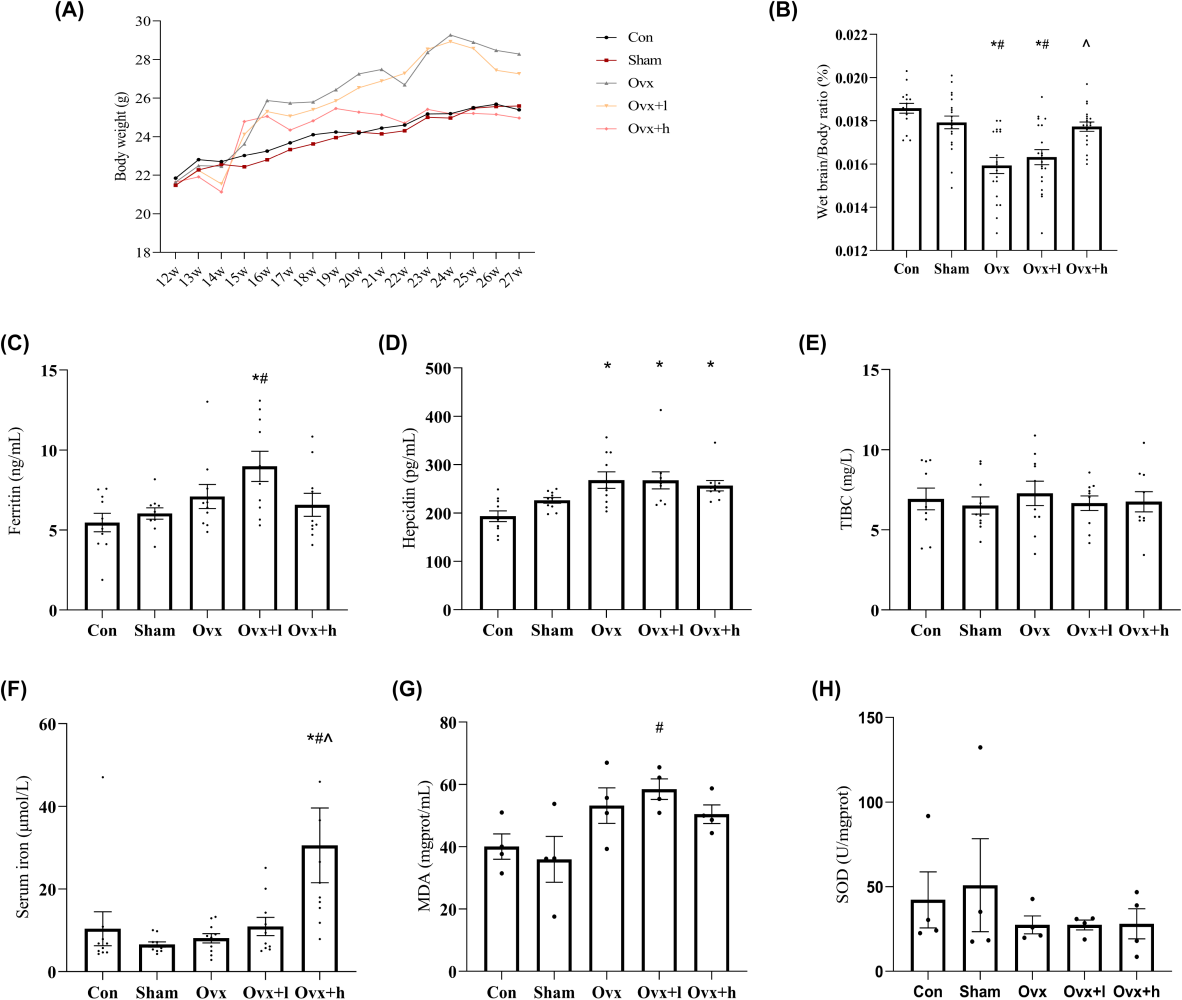
Body weight and serological tests of mice. (A) The changes in body weight of mice from 12 to 27 weeks. W, Week. (B) The radio of wet brain weight/body weight in different groups of mice. W, Week. (C–F) The levels of iron parameters comprised serum ferritin (*n* = 10), serum hepcidin (*n* = 10), TIBC (*n* = 10), and serum iron (*n* = 8). TIBC, Total iron binding capacity. (G, H) The levels of oxidative damage comprised MDA and SOD in mice (*n* = 4). Data were expressed as mean ± standard error of the mean (SEM). Data were analyzed using analysis of variance (ANOVA) with Tukey's post hoc test. Compared with Con mice, *p < 0.05. Compared with Sham mice, #*p* < 0.05. Compared with Ovx mice, ^*p* < 0.05.

The body weight of the mice was recorded from 12 to 27 weeks in order to observe the effect of iron accumulation on ovariectomized mice. In the 12th week, no difference occurred in body weight among the five groups (Figure 3A and Table S16). After ovariectomy and iron injection, the body weight of each group was different. Weight gain was faster in the Ovx and Ovx + l compared to other groups. Interestingly, the body weight in the Ovx + h increased at first but then remained relatively stable after 22 weeks. The results suggested that ovariectomy and iron accumulation affects the body weight of mice. Furthermore, the ratio of wet brain weight/body weight was measured after the mice were humanely sacrificed, which was decreased significantly in Ovx, Ovx + l, and Ovx + h groups (Figure 3B and Table S17).

### 3.4 Iron exposure affected iron content and caused oxidative damage in mice

To examine the effect of iron exposure on iron content and oxidative damage, we analyzed markers in the serum or hippocampus of mice. As expected, injection of iron caused serum ferritin, hepcidin, and serum iron concentrations to be elevated in Ovx + l and Ovx + h (all *p* < 0.05; Figure 3C–F and Table S18). Compared with the Sham mice, the final lipid peroxidation product MDA in Ovx, Ovx + l mice increased (*p* < 0.05; Figure 3G and Table S19), suggesting that the degree of lipid peroxidation in the hippocampus of mice was increased, and indirectly reflected the degree of cell damage was increased. No significant difference was found in SOD activity (Figure 3H and Table S19).

### 3.5 Iron exposure caused histopathological changes in the hippocampus of mice

**FIGURE 4:**
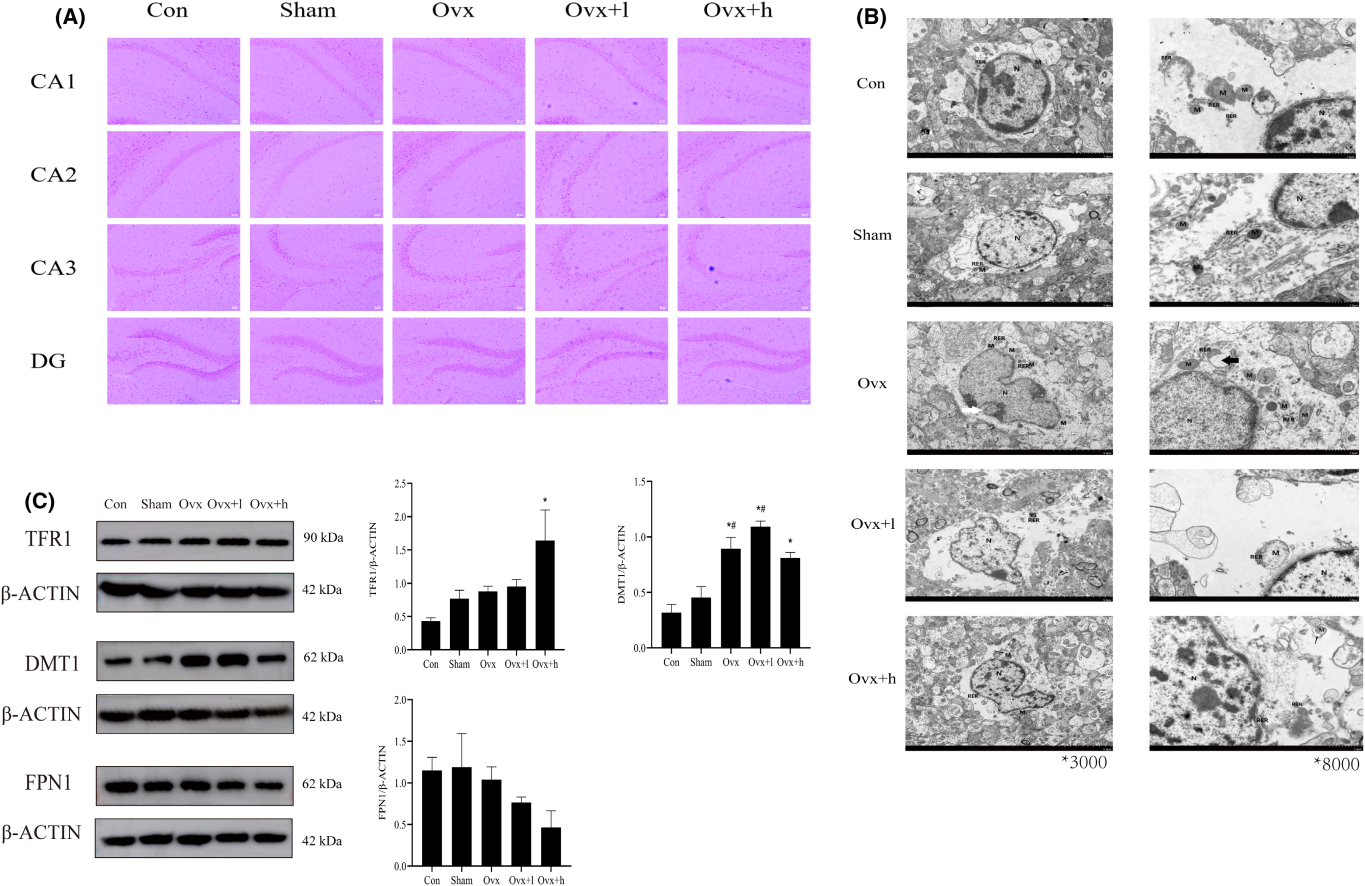
Histopathological changes in the hippocampus of mice. (A, B) Field of interest and representative images of Perl's staining and transmission electron microscopy (TEM) in brain sections after sacrifice. M, Mitochondrion; N, Nucleus; RER, Rough endoplasmic reticulum. Perl's staining scale bar: 20 μm. TEM scale bar:5 μm (left) and 1 μm (right). (C) The protein levels of TFR1, DMT1 and FPN1 were detected in the hippocampus of mice (*n* = 3). β‐Actin was used as an internal control, and the results showed a fold change in the control. Error bars indicate SEM. Compared with Con mice, *p < 0.05. Compared with Sham mice, #*p* < 0.05.

Perl's staining was used to detect the iron within the hippocampus cell, which was observed under 100× magnification (Figure 4A). Amazingly, a small amount of iron‐positive cells was observed in the hippocampus of the Ovx mice and a large number was observed in Ovx + l and Ovx + h mice. It was found that iron accumulation has an impact on hippocampal CA1, CA2, CA3, and DG regions, especially the DG region.

To further confirm that iron accumulation and ovariectomy changed the structure of the cell, ultrathin sections from the brain of mice procedure were prepared, and the ultrastructure of mice hippocampus was imaged by TEM (Figure 4B). Swelling of hippocampal cells and obvious shrunken mitochondria in Ovx, Ovx + l, and Ovx + h groups were observed. The number of cellular organs in cytoplasm was less, and there were fewer Golgi apparatus, mitochondria, granular endoplasmic reticulum, and ribosomes. Moreover, a marked deformation of the nucleus was found in Ovx, Ovx + l, and Ovx + h mice.

### 3.6 Differential expression of proteins in hippocampus

**FIGURE 5:**
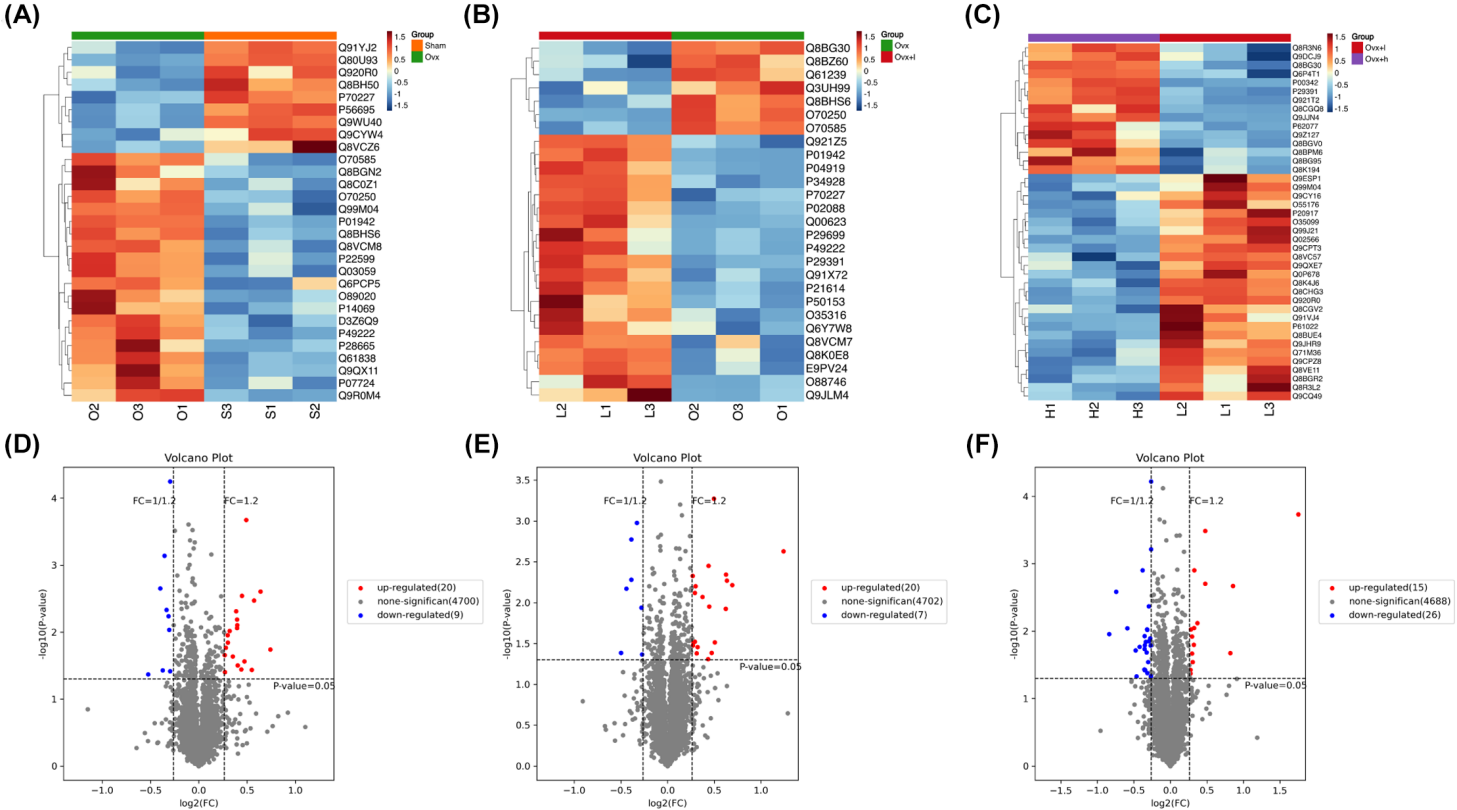
Differential expression of hippocampal proteins in different groups of mice (*n* = 3). (A–C) Heat map of altered protein expression in Ovx vs. Sham, Ovx + l vs. Ovx and Ovx + h vs. Ovx + l mice, respectively. S1‐3, O1‐3, L13, and H1‐3 represent mice of Sham, Ovx, Ovx + l, and Ovx + h, respectively. (D–F) Volcano plot for the identified proteins. Red and blue dots indicate significantly upregulated and downregulated proteins, respectively.

Based on the above results, we further performed the proteomics underlying iron overload in the hippocampus of ovariectomized mice. A total of 4729 proteins were detected by proteomics (criteria: unique polypeptide sequence ≥1, false discovery rate <1%). Among the 4729 proteins, 29/27/41 proteins were significantly differentially expressed in the hippocampus in Ovx vs. Sham, Ovx + l vs. Ovx, and Ovx + h vs. Ovx + l mice, respectively. The upregulation or downregulation proteins of each group were shown in the volcano plot (Figure 5). The specific information on differentially expressed proteins are shown in Figure 5.

**FIGURE 6:**
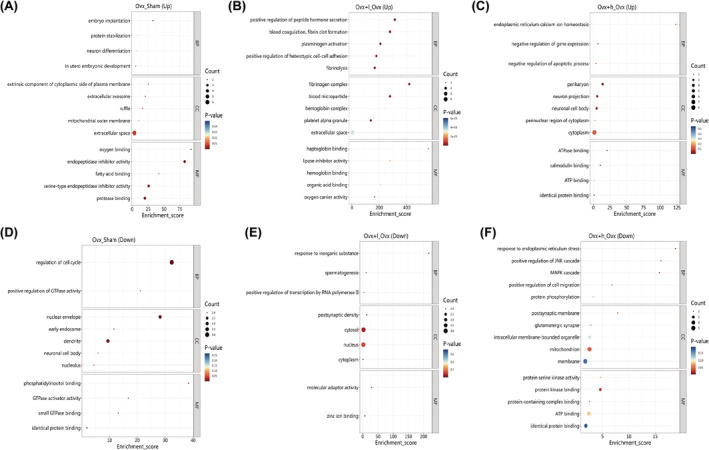
Gene Ontology (GO) enrichment analysis of differentially expressed proteins involved in the biological process (BP), cellular component (CC), and molecular function (MF) in Ovx vs. Sham, Ovx + l vs. Ovx, and Ovx + h vs. Ovx + l mice. X‐axis Enrichment Score was enrichment score, and Y‐axis was top 5 term information of BP/CC/MF, respectively. Entries with larger bubbles contain more protein. The color of bubbles varied from blue to red, and the smaller the enrichment pvalue, the greater the significance.

To categorize the differential expression of hippocampal proteins, gene ontology analysis was performed. The differentially expressed proteins in the hippocampus of Ovx vs. Sham, Ovx + l vs. Ovx and Ovx + h vs. Ovx + l mice were classified and enriched according to the BP, CC, and MF. The result for BP of the identified proteins mainly involved signal transduction, neuron differentiation, axon regeneration, protein stabilization, regulation of cell cycle, etc. The result for CC of the identified proteins mainly involved dendrite, mitochondrial outer membrane, extracellular space, nuclear envelope, fibrinogen complex, etc. The result for CC of the identified proteins mainly involved organic acid binding, hemoglobin binding, lipase inhibitor activity, molecular adaptor activity, identical protein binding, etc (Figure 6).

**FIGURE 7:**
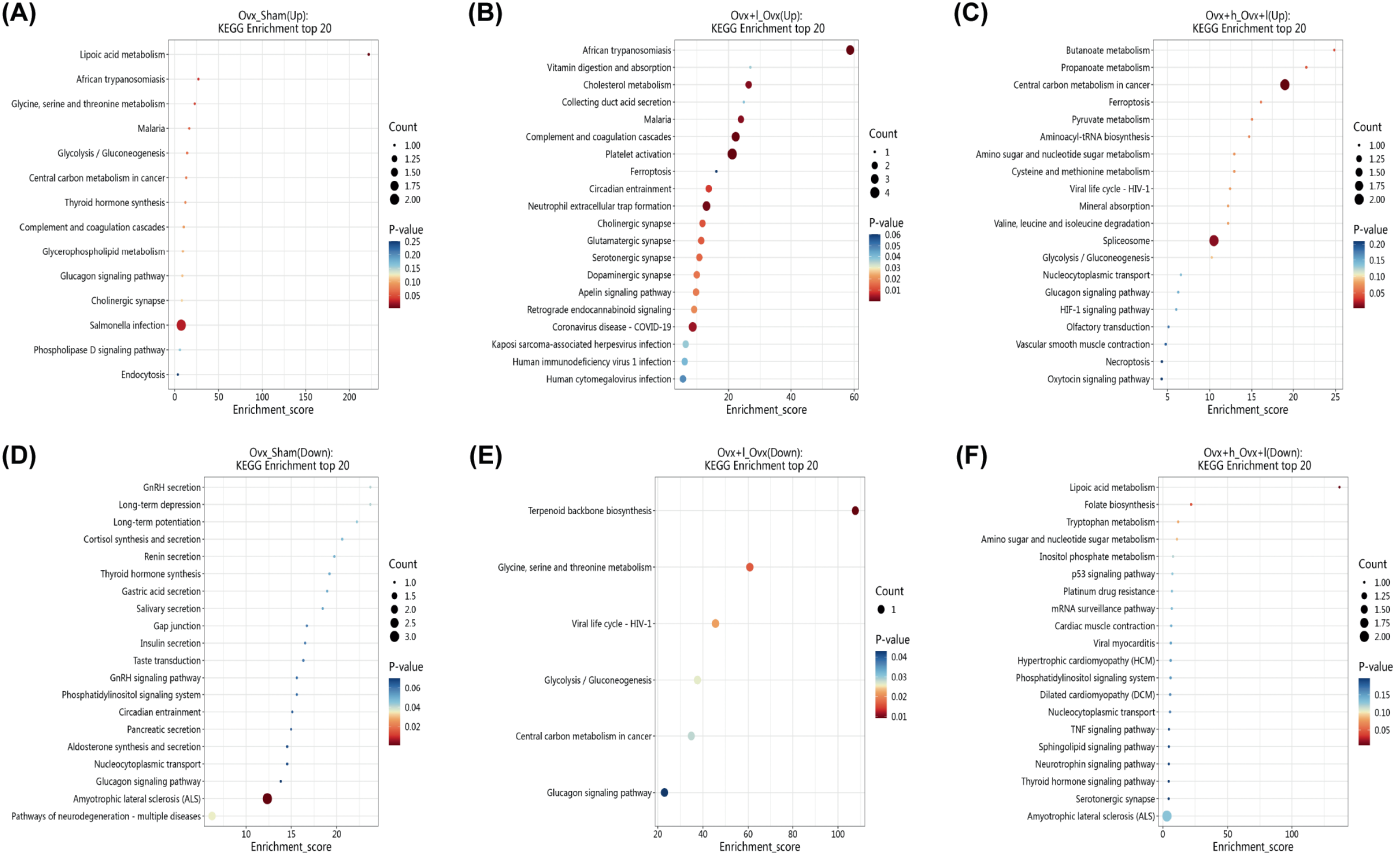
Kyoto Encyclopedia of Genes and Genomes (KEGG) pathway analysis of the dysregulated proteins in Ovx vs. Sham, Ovx + l vs. Ovx, and Ovx + h vs. Ovx + l mice. X‐axis Enrichment Score was enrichment score, and Y‐axis was pathway information of top 20. Entries with larger bubbles contain more protein. The color of bubbles varied from blue to red, and the smaller the enrichment *p* value, the greater the significance.

KEGG enrichment was performed for differentially expressed proteins of Ovx vs. Sham, Ovx + l vs. Ovx and Ovx + h vs. Ovx + l mice. Figure 7 showed the 20 most altered pathways included ferroptosis, cholinergic synapse, glycolysis/gluconeogenesis, phospholipase D signaling pathway, glucagon signaling pathway, pathways of neurodegeneration—multiple diseases, HIF‐1 signaling pathway, necroptosis, p53 signaling pathway, and TNF signaling pathway, etc.

Additionally, we also found that 78/84/76 proteins were significantly differentially expressed in the hippocampus among Ovx + h/Ovx + l/Ovx mice vs. Con mice. The specific information of differentially expressed proteins was shown in Tables S20–S22. KEGG enrichment was performed for differentially expressed proteins between Ovx + h/Ovx + l/Ovx mice and the Con mice, which were closely linked to nervous system activity including metabolism of xenobiotics by cytochrome P450, Wnt signaling pathway, PPAR signaling pathway, AMPK signaling pathway, mTOR signaling pathway, MAPK signaling pathway, PI3K‐Akt signaling pathway, inflammatory mediator regulation of TRP channels, dopaminergic synapse, and cAMP signaling pathway, etc.

To further validate the proteomics results and association with iron and ovariectomized mice, western blot was performed to detect the expression levels of TFR1, DMT1, and FPN1 in the hippocampus of mice. In comparison with Sham and Ovx, the expression of TFR1 and DMT1 significantly increased obviously after intraperitoneal injection of FAC (all *p* < 0.05). The expression of FPN1 was not significantly decreased in ovariectomized mice compared to that in the Con mice, but added FAC showed a dose‐dependent decrease in ovariectomized mice.

## 4 DISCUSSION

The present study aimed to assess the effects and mechanisms of iron accumulation on cognitive disorders in ovariectomized mice. We constructed ovariectomized model by giving C57BL/6 mice bilateral ovariectomy. Previous studies using ovariectomized mice did not observe excess iron in animal serum iron levels. Therefore, ovariectomized mice were treated with FAC to mimic iron accumulation in postmenopausal women.^24^ Subsequent studies in OFT, EPM, TST, FST, MWM and light/dark transition tests showed that iron accumulation had negative effects on cognitive function in ovariectomized mice. Then, the iron accumulation in serum and hippocampus of mice was verified. TEM showed that swelling of hippocampal cells and obvious atrophy of mitochondria. Finally, a wide range of molecular changes was revealed by TMT proteomics analysis of differential proteins and pathway enrichment in different groups of mice. The downregulation of TFR1 and DMT1, the significant iron homeostasis proteins, was further verified by western blot in the iron‐treated mice. Therefore, we concluded that iron accumulation could have a negative effect on ovx‐induced cognitive disorders.

Analysis of data obtained from behavioral tests consistently showed that iron accumulation induced cognitive impairment in mice. A study of Wistar rat pups on days 10–12, behavioral tests included OFT and radial arm maze demonstrated deficits in acquisition performance after administration of Fe^2+^ at doses of either 2.5, 7.5, 15.0 or 30.0 mg/kg.^25^ In line with the precent study, a series of behavioral tests of Ovx, Ovx + l, and Ovx + h mice showed obvious anxiety/depression‐like behavior, learning and memory defects in a dose‐dependent manner. In addition, deferoxamine is a potent iron chelator, which was delivered to HD mice over the 2‐week dosing and showed gradual improvement in endurance in accelerating rota‐rod test.^26^

The menopausal age of Chinese women is roughly 50 years.^27^ Normally, the older the age and the longer the menopause, the lower the estrogen level in the body. Our study used mice in which ovaries were removed to mimic women without menses and FAC was injected into the Ovx + l/Ovx + h mice to mimic iron accumulation in postmenopausal women. As expected, the mice differed in the serum iron parameters in our experiments. Due to there being no physiological processes to excrete excess iron, ferritin acts as an iron storage protein throughout the body. Its main role is in iron sequestration in which it functions as a ferricoxidase, converting Fe^2+^ to Fe^3+^ as iron is internalized and sequestered in the ferritin mineral core.^28^ In cases of iron deficiency and overload, serum ferritin is regarded as the most sensitive and specific of the various blood tests available for diagnosis.^29^ Hepcidin is a liver‐derived regulatory hormone that binds to the ferroportin on the cell surface, inducing its internalization and degradation and preventing cellular iron export.^30^, ^31^ A strong relationship between ferritin/hepcidin and NDs had documented in plenty of literature.^31^, ^32^, ^33^ A similar conclusion was obtained from the present results. Meanwhile, our findings showed that the level of serum iron in Ovx + h mice was increased, which was consistent with the findings proposed by Kweon et al.^32^ Moreover, a small amount of iron deposition was observed in the Ovx mice, and a large amount of iron deposition was observed in the Ovx + l and Ovx + h mice in different regions of the hippocampus, which proved that iron caused damage to the brain. Through the observation of ultrastructure, it was found that the cell shape of Ovx, Ovx + l, and Ovx + h mice was changed, and the cytoplasm such as mitochondria and Golgi apparatus was damaged obviously.

Hippocampal proteome analysis revealed that differentially expressed proteins were mainly enriched at negative regulation of neuron projection development, mitochondrial outer membrane, regulation of postsynaptic neurotransmitter receptor internalization, dendritic spine, glutamatergic synapse, postsynaptic density membrane, endoplasmic reticulum calcium ion homeostasis, etc. When iron overload occurs in cells, iron with high redox activity induces ferroptosis by participating in the Fenton and Haber–Weiss chemical reactions to produce ROS.^34^ Deterioration of synaptic function, mitochondrial dysfunction, and loss of dendrites and axons are pathological features of neurodegenerative diseases such as AD and HD.^35^ A clinical study showed that atrophy of the cholinergic basal forebrain occurs during aging and that increased atrophy was found in patients with cognitive dysfunction.^36^ Choline acetyltransferase (CHAT) was found to be downregulated in the Ovx rat model, which was consistent with our proteomic findings.^37^ ChAT can synthesize acetylcholine (Ach) by mediating the transfer of acetyl groups from acetyl coenzyme A to choline at synaptic terminals of cholinergic neurons. Ach, as a cholinergic neurotransmitter, is released from a wide range of cortical and subcortical sites at synaptic terminals and plays an important role in cognitive functions such as learning and memory.^38^ Meanwhile, the expression of vasoactive intestinal polypeptide (VIP) and CHAT are considered VIP/ChAT interneurons, which are one of the main sources of cholinergic input to the mammalian cortex.^39^

Mitochondria damage in the hippocampus of mice was also found in our study. We found that iron treatment led to a decrease in the mitochondrial protein Akap1, a neuroprotective mitochondrially oriented scaffold protein that binds to the holo enzymatic form of PKA in hippocampal neurons. In neurons, they can promote mitochondrial interconnection, anterograde mitochondrial transport in dendrites, and mitophagy.^40^, ^41^ Mechanistically, mitochondrial PKA phosphorylates the mitochondrial fission modulator Drp1 on serine 637 to inhibit mitochondrial fission and promote neuroprotection against glutamate excitotoxicity and oxidative stress in a cell culture model of Parkinson's disease (PD).^42^, ^43^ Increasing evidence suggested that the damage of PKA/CREB pathway may be one of the causes of cognitive impairment.^44^, ^45^

In addition, our study also identified several pathways associated with cognitive decline as described previously. AMP‐activated protein kinase (AMPK) is a major regulator of cellular energy homeostasis.^46^ A recent study showed that energy stress‐activated AMPK and inhibited ACC, leading to inhibition of fatty acid synthesis and ferroptosis inhibition.^47^ Decreased AMPK activity in the brains of AD patients was found in previous studies,^48^, ^49^ which may be since AMPK is a key player in the defects caused by Aβ oligomers and any decrease in its activity would negatively affect the AD brain.^50^ Furthermore, hypometabolism due to decreased glucose uptake has also been suggested as a key abnormality in the early stages of AD.^51^ It has been shown that AMPK activation is responsible for promoting glucose uptake and improving brain energy metabolism because it regulates transport mechanisms and protects cells from hypoxic damage.^52^, ^53^ Previous studies found that hypoxia‐inducible factor‐1 (HIF‐1) impairs could lead to decreased glucose uptake in the brain.^54^

In our study, we experimented western blot test on key proteins related to iron. For circulating iron to enter the brain, it must first through the blood brain barrier in the microvascular endothelial cells.^55^ Ferrous and iron then enter the cytoplasm via DMT1 and TFR1, respectively. FPN1, as the only known iron exporter, is downregulated with age in AD mouse models and AD patients.^56^, ^57^ Our results found that iron deposition in ovariectomized mice owed to a decline in the TfR1 and DMT1 expression and a rise in the FPN1 expression. This is consistent with the conclusions of previous studies.^58^, ^59^

## 5 CONCLUSION

In conclusion, we reported that iron accumulation mice model with ovariectomy could lead to the decline of movement and cognitive function. Iron exposure could cause histopathological damage in the hippocampus of ovariectomized mice and, by disturbing hippocampus proteome, particularly the expression of hippocampal iron metabolism‐related proteins, could further influence cognitive impairment in ovariectomized mice. Our results might provide new insights into the molecular mechanisms of neurotoxicology caused by iron exposure in ovariectomized mice and might offer a new perspective on preventing cognitive decline in postmenopausal women. In addition, as trace element supplementation becomes increasingly normalized, our results might provide guidance for iron supplementation in postmenopausal women.

## FUNDING INFORMATION

This work was supported by research funds from the National Natural Science Foundation of China (Grant No. 82003478) and the Scientific and Technological Project in Henan Province (Grant No. 242102310031).

## CONFLICT OF INTEREST STATEMENT

The author declares that they have no conflict of interest.

## Supporting information


Appendix S1

## Data Availability

Research data are not shared.
