# Supplementary material for: Effect of ferric citrate on hippocampal iron accumulation and widespread molecular alterations associated with cognitive disorder in an ovariectomized mice model
Source: CNS Neurosci Ther. 2024 Sep 9;30(9):e70018. doi: 10.1111/cns.70018 (PMC11386256; doi:10.1111/cns.70018)
Supplement: Supplementary file 1 — Appendix S1 [file CNS-30-e70018-s001.docx]

**Appendices**

**
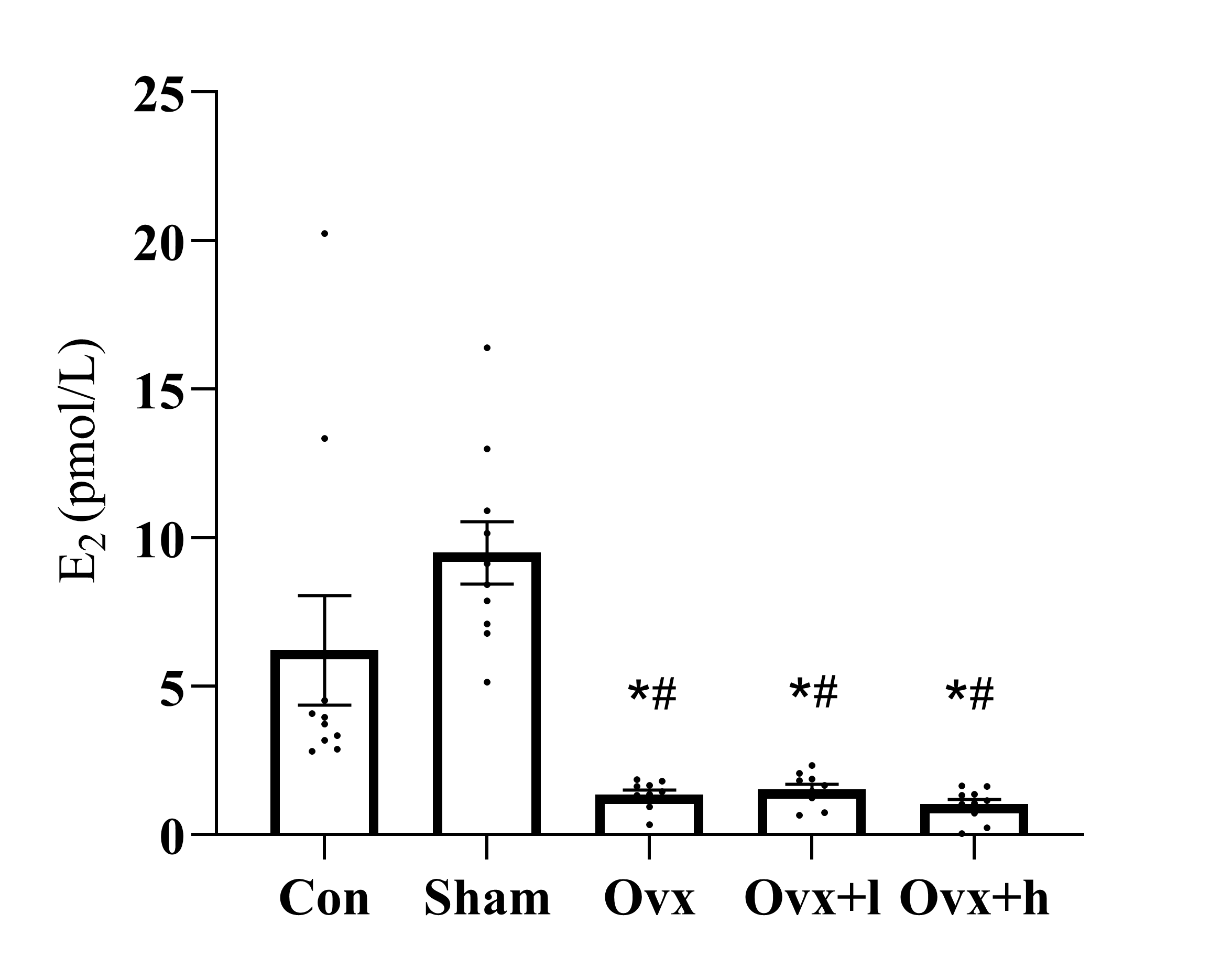
**

Figure S1 Serum estradiol (E_2_) levels in different groups of mice (n=10).

Table S1 Serum estradiol levels (pmol/L) in different groups of mice

| Group | Mean | SD | *F* | *P* |
| --- | --- | --- | --- | --- |
| Con | 6.22 | 5.83 | 0.781 | 0.540 |
| Sham | 9.49 | 3.31 |  |  |
| Ovx | 1.36 | 0.46 |  |  |
| Ovx+l | 1.53 | 0.54 |  |  |
| Ovx+h | 1.03 | 0.54 |  |  |

Table S2 The distance moved, mean velocity and immobility time in total, center and side areas respectively in the open field test

| Group | Distance moved (mm) | | |  | Mean velocity (mm/s) | | |  | Immobility time (s) | | |
| --- | --- | --- | --- | --- | --- | --- | --- | --- | --- | --- | --- |
|  | Total | Center | Side |  | Total | Center | Side |  | Total | Center | Side |
| Con | 19780.63±5138.90 | 1459.73±887.78 | 18320.91±4937.98 |  | 66.01±17.18 | 64.96±26.77 | 66.59±17.72 |  | 86.37±39.60 | 5.30±10.35 | 81.08±39.41 |
| Sham | 20579.29±4382.29 | 1456.94±761.41 | 19122.35±3809.14 |  | 68.82±14.77 | 78.05±39.07 | 69.51±15.29 |  | 82.23±20.81 | 5.87±10.31 | 76.23±23.06 |
| Ovx | 16527.41±5402.29 | 1321.90±712.33 | 15205.51±5104.96 |  | 55.17±18.04 | 60.47±39.38 | 56.01±16.71 |  | 108.21±40.32 | 12.99±22.45 | 95.22±34.96 |
| Ovx+l | 14587.52±5215.67 | 944.42±682.95 | 13643.09±4904.11 |  | 48.86±17.53 | 68.66±44.77 | 49.62±16.73 |  | 124.62±41.45 | 15.61±40.66 | 109.01±35.06 |
| Ovx+h | 12851.83±4073.48 | 829.66±456.21 | 12022.18±3879.96 |  | 43.05±13.58 | 67.17±27.54 | 42.64±13.39 |  | 130.76±38.17 | 5.85±18.05 | 124.83±37.35 |
| *F* | 8.907 | 3.377 | 8.454 |  | 8.780 | 0.524 | 9.637 |  | 6.787 | 0.798 | 6.633 |
| *P* | <0.001 | 0.013 | <0.001 |  | <0.001 | 0.718 | <0.001 |  | <0.001 | 0.529 | <0.001 |

Table S3 The distance moved in total, center, open and close areas respectively in the elevated plus maze test

| Group | Distance moved (mm) | | | |
| --- | --- | --- | --- | --- |
|  | Total | Center | Open arm | Close arm |
| Con | 17367.47±3155.18 | 410.55±314.83 | 443.10±712.83 | 16510.18±3324.31 |
| Sham | 18857.42±4841.01 | 776.98±1046.52 | 803.75±689.78 | 17261.43±3852.81 |
| Ovx+l | 16191.15±3986.80 | 328.59±234.57 | 949.57±1014.16 | 14907.22±3794.36 |
| Ovx+h | 13101.35±5388.79 | 504.43±384.89 | 709.09±873.18 | 11887.82±5211.23 |
| *F* | 5.828 | 2.290 | 0.806 | 6.772 |
| *P* | <0.001 | 0.066 | 0.524 | <0.001 |

Table S4 The mean velocity in total, center, open and close areas respectively in the elevated plus maze test.

| Group | Mean velocity (mm/s) | | | |
| --- | --- | --- | --- | --- |
|  | Total | Center | Open arm | Close arm |
| Con | 57.93±10.53 | 46.33±22.04 | 14.56±15.46 | 63.65±9.18 |
| Sham | 63.90±17.86 | 55.83±38.52 | 30.42±19.73 | 71.15±18.09 |
| Ovx | 61.99±11.48 | 45.41±23.65 | 40.88±67.29 | 66.66±11.69 |
| Ovx+l | 54.09±13.31 | 39.56±14.00 | 43.72±57.55 | 59.70±19.37 |
| Ovx+h | 43.98±18.30 | 40.40±24.50 | 24.28±23.32 | 50.50±21.70 |
| *F* | 5.618 | 1.251 | 1.400 | 4.214 |
| *P* | <0.001 | 0.295 | 0.241 | 0.004 |

Table S5 The immobility time in total, center, open and close areas respectively in the elevated plus maze test.

| Group | Immobility time (s) | | | |
| --- | --- | --- | --- | --- |
|  | Total | Center | Open arm | Close arm |
| Con | 87.83±21.23 | 3.70±4.02 | 8.55±16.76 | 75.42±24.91 |
| Sham | 81.93±24.75 | 3.61±3.97 | 5.03±4.47 | 73.13±25.74 |
| Ovx | 79.07±24.61 | 2.73±2.97 | 5.53±7.17 | 70.81±26.50 |
| Ovx+l | 96.01±35.75 | 2.54±2.34 | 12.55±22.86 | 80.87±40.86 |
| Ovx+h | 121.77±47.92 | 3.47±4.85 | 10.01±23.42 | 108.29±50.50 |
| *F* | 5.381 | 0.387 | 0.679 | 3.627 |
| *P* | 0.001 | 0.818 | 0.608 | 0.009 |

Table S6 The time of enter target area included center, open and close areas respectively in the elevated plus maze test.

| Group | Time of enter target area | | |
| --- | --- | --- | --- |
|  | Center | Open arm | Close arm |
| Con | 2.69±2.18 | 0.75±0.77 | 6.94±3.43 |
| Sham | 4.20±3.62 | 2.00±1.65 | 8.45±3.28 |
| Ovx | 1.84±1.17 | 1.63±1.54 | 9.68±4.00 |
| Ovx+l | 2.35±1.76 | 1.75±1.21 | 8.10±3.89 |
| Ovx+h | 2.60±2.33 | 1.35±1.39 | 5.60±3.38 |
| *F* | 2.728 | 2.141 | 3.569 |
| *P* | 0.034 | 0.082 | 0.009 |

Table S7 The percentage of immobility time, struggling time and climbing time in the tail suspension test.

| Group | Immobility time (%) | Struggling time(%) | Climbing time(%) |
| --- | --- | --- | --- |
| Con | 40.80±25.29 | 30.73±23.04 | 28.45±26.66 |
| Sham | 36.36±19.52 | 29.21±23.04 | 34.40±26.59 |
| Ovx | 46.54±23.46 | 38.77±24.46 | 12.53±10.71 |
| Ovx+l | 48.86±18.68 | 32.03±19.75 | 18.95±21.32 |
| Ovx+h | 51.23±14.29 | 36.25±20.76 | 12.24±13.27 |
| *F* | 1.748 | 0.615 | 4.400 |
| *P* | 0.146 | 0.663 | 0.003 |

Immobility was defined as when the mouse stopped struggling and the body remained vertically upside down. Struggle was considered to be a struggle movement that was clearly visible in mice. Climbing was considered to be a powerful and vigorous movement of the mouse's limbs up and down.

immobility time (%) indicated percentage of immobility time. Struggling time(%) indicated percentage of struggling time. Climbing time(%) indicated percentage of climbing time.

Table S8 The percentage of immobility time, swimming time and struggling time in the forced-swimming test.

| Group | Immobility time (%) | Swimming time(%) | Struggling time(%) |
| --- | --- | --- | --- |
| Con | 33.07±23.07 | 49.82±17.40 | 17.09±20.94 |
| Sham | 46.20±18.58 | 46.54±15.95 | 7.24±5.87 |
| Ovx | 49.61±17.50 | 41.70±14.22 | 8.67±6.55 |
| Ovx+l | 52.31±21.82 | 40.74±17.56 | 6.93±6.20 |
| Ovx+h | 54.42±16.92 | 40.42±15.86 | 5.14±5.05 |
| *F* | 3.171 | 1.167 | 3.624 |
| *P* | 0.017 | 0.331 | 0.009 |

Table S9 The latency of C57BL/6 mice in the morris water maze training trials

| Group | Latency (s) | | | | |
| --- | --- | --- | --- | --- | --- |
|  | Day 1 | Day 2 | Day 3 | Day 4 | Day 5 |
| Con | 51.90±11.08 | 42.00±20.60 | 39.05±20.37 | 34.69±18.98 | 27.59±19.61 |
| Sham | 55.48±8.46 | 42.56±19.80 | 32.08±19.92 | 30.49±20.61 | 28.34±20.31 |
| Ovx | 54.14±11.95 | 39.65±20.75 | 36.36±21.54 | 37.22±20.90 | 29.75±18.68 |
| Ovx+l | 58.09±3.69 | 42.79±19.93 | 35.25±21.88 | 30.95±19.92 | 27.54±20.79 |
| Ovx+h | 57.69±6.54 | 49.12±17.03 | 49.46±17.51 | 48.59±18.13 | 44.79±20.42 |

Table S10 The swimming speed of C57BL/6 mice in the morris water maze training trials

| Group | Swimming speed (mm/s) | | | | |
| --- | --- | --- | --- | --- | --- |
|  | Day 1 | Day 2 | Day 3 | Day 4 | Day 5 |
| Con | 141.43±26.38 | 152.13±46.45 | 139.96±40.90 | 135.69±49.75 | 141.66±37.50 |
| Sham | 146.14±24.91 | 165.74±47.16 | 137.31±41.94 | 136.90±34.50 | 137.03±39.31 |
| Ovx | 146.15±35.06 | 152.87±47.11 | 142.43±47.50 | 145.41±42.15 | 133.90±41.79 |
| Ovx+l | 122.94±26.17 | 138.08±45.50 | 135.20±47.57 | 133.90±43.26 | 130.41±35.20 |
| Ovx+h | 113.38±24.27 | 117.87±45.31 | 100.09±46.79 | 90.11±43.68 | 94.94±49.47 |

Table S11 The crossing number and percent of time spent in the platform quadrant in the probe trial of the morris water maze test.

| Group | Crossing number (count) | Percent of time spent in the platform quadrant（%） |
| --- | --- | --- |
| Con | 7.06±2.83 | 18.28±9.12 |
| Sham | 5.15±0.94 | 16.57±9.61 |
| Ovx | 4.84±2.95 | 13.61±7.52 |
| Ovx+l | 4.90±3.09 | 16.45±10.30 |
| Ovx+h | 1.85±2.35 | 8.88±12.64 |
| *F* | 8.023 | 2.569 |
| *P* | <0.001 | 0.043 |

Table S12 The 1 hour tested in light chamber in the light/dark transition test.

| Group | Latency (s) | Error times | L-time (s) | L-time% |
| --- | --- | --- | --- | --- |
| Con | 168.84±104.86 | 3.94±3.99 | 259.43±52.15 | 86.47±17.38 |
| Sham | 171.97±104.75 | 3.47±2.93 | 254.71±49.16 | 84.89±16.39 |
| Ovx | 137.17±98.69 | 5.00±4.04 | 227.06±56.14 | 75.68±18.71 |
| Ovx+l | 138.84±118.43 | 5.15±4.88 | 226.44±68.81 | 75.47±22.94 |
| Ovx+h | 73.54±74.87 | 7.55±8.76 | 167.65±77.67 | 55.87±25.88 |
| *F* | 2.933 | 1.646 | 6.542 | 6.543 |
| *P* | 0.025 | 0.17 | <0.001 | <0.001 |

Table S13 The 1 hour tested in dark chamber in the light/dark transition test.

| Group | D-time (s) | D-time% |
| --- | --- | --- |
| Sham | 45.25±49.18 | 15.08±16.39 |
| Con | 40.53±52.17 | 13.51±17.38 |
| Ovx | 72.90±56.14 | 24.30±18.71 |
| Ovx+l | 73.52±68.82 | 24.50±22.94 |
| Ovx+h | 117.30±72.36 | 39.09±24.12 |
| *F* | 4.746 | 4.474 |
| *P* | 0.002 | 0.002 |

Table S14 The 24 hour tested in light chamber of C57BL/6 mice in the light/dark transition test.

| Group | Latency (s) | Error times | L-time (s) | L-time% |
| --- | --- | --- | --- | --- |
| Con | 148.30±129.27 | 5.31±5.53 | 257.94±59.85 | 85.97±19.74 |
| Sham | 138.03±125.85 | 4.35±4.33 | 238.38±65.23 | 79.45±21.74 |
| Ovx | 110.56±99.15 | 5.68±3.70 | 213.16±74.17 | 71.04±24.72 |
| Ovx+l | 125.59±112.28 | 5.60±4.06 | 226.21±57.13 | 75.39±19.04 |
| Ovx+h | 33.75±68.89 | 6.05±3.02 | 138.59±87.21 | 46.19±29.06 |
| *F* | 3.409 | 0.479 | 8.151 | 8.153 |
| *P* | 0.012 | 0.751 | <0.001 | <0.001 |

Table S15 The 24 hour tested in dark chamber of C57BL/6 mice in the light/dark transition test.

| Group | D-time (s) | D-time% |
| --- | --- | --- |
| Con | 42.01±59.85 | 14.00±19.95 |
| Sham | 61.57±65.23 | 20.52±21.74 |
| Ovx | 86.80±74.18 | 28.93±24.72 |
| Ovx+l | 73.75±57.12 | 24.58±19.04 |
| Ovx+h | 161.38±87.21 | 53.78±29.06 |
| *F* | 8.153 | 8.151 |
| *P* | <0.001 | <0.001 |

Table S16 The changes in body weight of C57BL/6 mice from 10 to 27 weeks

| Variable | Con | Sham | Ovx | Ovx+l | Ovx+h |
| --- | --- | --- | --- | --- | --- |
| 10w | 19.69±2.09 | 20.26±0.56 | 20.25±0.95 | 20.39±0.65 | 20.36±0.64 |
| 11w | 21.75±0.88 | 21.83±0.71 | 21.86±1.03 | 21.50±0.77 | 21.54±0.75 |
| 12w | 21.85±0.88 | 21.48±0.52 | 21.65±0.92 | 21.49±0.74 | 21.65±1.00 |
| 13w | 22.81±0.99 | 22.28±0.60 | 22.50±0.99 | 22.25±0.88 | 21.92±1.14 |
| 14w | 22.71±1.23 | 22.56±0.73 | 22.47±0.90 | 21.55±0.93 | 21.13±3.14 |
| 15w | 23.03±1.28 | 22.44±0.94 | 23.63±0.90 | 24.12±1.31 | 24.79±1.41 |
| 16w | 23.26±1.63 | 22.80±0.55 | 25.88±1.40 | 25.31±1.53 | 25.06±1.55 |
| 17w | 23.69±1.48 | 23.34±0.80 | 25.75±1.40 | 25.07±1.19 | 24.35±1.05 |
| 18w | 24.11±1.31 | 23.63±0.78 | 25.81±1.46 | 25.41±1.39 | 24.83±1.15 |
| 19w | 24.25±1.26 | 23.96±0.73 | 26.44±1.82 | 25.86±2.41 | 25.47±1.25 |
| 20w | 24.19±0.81 | 24.24±0.89 | 27.26±2.09 | 26.54±2.20 | 25.28±1.22 |
| 21w | 24.45±0.88 | 24.15±0.75 | 27.49±2.36 | 26.89±2.24 | 25.14±1.26 |
| 22w | 24.61±0.91 | 24.31±1.07 | 26.69±2.69 | 27.29±2.50 | 24.71±1.30 |
| 23w | 25.18±1.04 | 25.01±0.77 | 28.37±2.99 | 28.54±2.83 | 25.42±1.14 |
| 24w | 25.20±1.32 | 24.97±0.80 | 29.28±3.55 | 28.93±3.00 | 25.20±1.02 |
| 25w | 25.51±0.90 | 25.48±1.05 | 28.90±3.28 | 28.57±2.75 | 25.21±0.91 |
| 26w | 25.69±0.99 | 25.57±0.87 | 28.48±2.48 | 27.45±2.51 | 25.16±1.06 |
| 27w | 25.40±1.01 | 25.60±1.05 | 28.29±2.44 | 27.27±2.02 | 24.97±1.12 |

Table S17 The radio of brain wet weight/body weight at 27 months of age

| Group | Mean | SD | *F* | *P* |
| --- | --- | --- | --- | --- |
| Con | 0.0186 | 0.0009 | 13.457 | <0.001 |
| Sham | 0.0179 | 0.0013 |  |  |
| Ovx | 0.0159 | 0.0016 |  |  |
| Ovx+l | 0.0163 | 0.0016 |  |  |
| Ovx+h | 0.0177 | 0.0009 |  |  |

Table S18 The levels of iron parameters comprised serum ferritin, serum hepcidin, serum iron, and TIBC

|  | Con | Sham | Ovx | Ovx+l | Ovx+h | *F* | *P* |
| --- | --- | --- | --- | --- | --- | --- | --- |
| Ferritin ng/mL  （n=10） | 5.47±0.58 | 6.03±0.35 | 7.10±0.75 | 8.98±0.94 | 6.57±0.72 | 3.720 | 0.011 |
| Hepcidin pg/mL（n=10） | 193±34.85 | 226±18.57 | 268±54.22 | 268±56.12 | 257±33.89 | 5.911 | <0.001 |
| Serum iron  umol/L  （n=8） | 5.75±0.46 | 5.75±0.75 | 9.26±1.05 | 12.40±2.5 | 24.11±4.15 | 11.598 | <0.001 |
| TIBC mg/L  （n=10） | 7.69±0.56 | 6.78±0.59 | 7.25±0.92 | 6.43±0.51 | 6.31±0.60 | 0.220 | 0.926 |

Table S19 The levels of oxidative damage comprised SOD and MDA in mice

|  | Con | Sham | Ovx | Ovx+l | Ovx+h | *F* | *P* |
| --- | --- | --- | --- | --- | --- | --- | --- |
| SOD U/mgprot  （n=4） | 42.25±16.61 | 50.92±27.47 | 27.45±5.29 | 27.44±2.91 | 28.13±8.91 | 0.509 | 0.730 |
| MDA mgprot/ml  （n=4） | 40.07±4.08 | 35.96±7.39 | 53.21±5.73 | 58.47±3.30 | 50.44±3.00 | 3.529 | 0.032 |

Table S20 The differentially expressed proteins in Ovx vs. sham mice

| Protein hydrolysates | Full name of protein | Item | Ovx vs. Sham | |
| --- | --- | --- | --- | --- |
|  |  |  | Ratio | P |
| Wfs1 | Wolframin | P56695 | 0.758 | 0.002 |
| Snx4 | Sorting nexin-4 | Q91YJ2 | 0.805 | 0.006 |
| Hdhd3 | Haloacid dehalogenase-like hydrolase domain-containing protein 3 | Q9CYW4 | 0.813 | 0.039 |
| Itpr3 | Inositol 1,4,5-trisphosphate receptor type 3 | P70227 | 0.794 | 0.005 |
| Lemd3 | Inner nuclear membrane protein Man1 | Q9WU40 | 0.781 | 0.001 |
| Als2 | Alsin | Q920R0 | 0.771 | 0.037 |
| Nup214 | Nuclear pore complex protein Nup214 | Q80U93 | 0.814 | 0.000 |
| Pch | Protein C18orf25 homolog | Q8BH50 | 0.808 | 0.009 |
| Sgsm3 | Small G protein signaling modulator 3 | Q8VCZ6 | 0.695 | 0.043 |
| Alb | Albumin | P07724 | 1.387 | 0.027 |
| Pzp | Pregnancy zone protein | Q61838 | 1.486 | 0.003 |
| Hba | Hemoglobin subunit alpha | P01942 | 1.404 | 0.000 |
| Pgam2 | Phosphoglycerate mutase 2 | O70250 | 1.307 | 0.005 |
| Mff | Mitochondrial fission factor | Q6PCP5 | 1.206 | 0.040 |
| Mug1 | Murinoglobulin-1 | P28665 | 1.275 | 0.023 |
| Serpina1b | Alpha-1-antitrypsin 1-2 | P22599 | 1.671 | 0.018 |
| Ncln | Nicalin | Q8VCM8 | 1.316 | 0.009 |
| Dtnb | Dystrobrevin beta | O70585 | 1.230 | 0.011 |
| Bin2 | Bridging integrator 2 | D3Z6Q9 | 1.318 | 0.008 |
| Cyth1 | Cytohesin-1 | Q9QX11 | 1.214 | 0.017 |
| Lias | Lipoyl synthase, mitochondrial | Q99M04 | 1.247 | 0.010 |
| Chat | Choline O-acetyltransferase | Q03059 | 1.232 | 0.014 |
| Armcx3 | Armadillo repeat-containing X-linked protein 3 | Q8BHS6 | 1.557 | 0.002 |
| D3Ertd751e | UPF0462 protein C4orf33 homolog | Q8BGN2 | 1.323 | 0.032 |
| Podxl | Podocalyxin | Q9R0M4 | 1.317 | 0.007 |
| Afm | Afamin | O89020 | 1.205 | 0.022 |
| S100a6 | Protein S100-A6 | P14069 | 1.463 | 0.037 |
| Fam234a | Protein FAM234A | Q8C0Z1 | 1.356 | 0.036 |
| Epb42 | Protein 4.2 | P49222 | 1.362 | 0.003 |

Table S21 The differentially expressed proteins in Ovx+l vs. Ovx mice

| Protein hydrolysates | Full name of protein | Item | Ovx+l vs. Ovx | |
| --- | --- | --- | --- | --- |
|  |  |  | Ratio | *P* |
| Pgam2 | Phosphoglycerate mutase 2 | O70250 | 0.796 | 0.001 |
| Shisa6 | Protein shisa-6 | Q3UH99 | 0.828 | 0.043 |
| Dtnb | Dystrobrevin beta | O70585 | 0.764 | 0.002 |
| Ston2 | Stonin-2 | Q8BZ60 | 0.708 | 0.041 |
| Fnta | Protein farnesyltransferase/geranylgeranyltransferase type-1 subunit alpha | Q61239 | 0.823 | 0.012 |
| Armcx3 | Armadillo repeat-containing X-linked protein 3 | Q8BHS6 | 0.737 | 0.007 |
| Nelfa | Negative elongation factor A | Q8BG30 | 0.763 | 0.005 |
| Hbb-b1 | Hemoglobin subunit beta-1 | P02088 | 1.552 | 0.005 |
| Hba | Hemoglobin subunit alpha | P01942 | 1.410 | 0.001 |
| Apoa1 | Apolipoprotein A-I | Q00623 | 1.294 | 0.009 |
| Fgb | Fibrinogen beta chain | Q8K0E8 | 1.539 | 0.012 |
| Fga | Fibrinogen alpha chain | E9PV24 | 1.615 | 0.006 |
| Tom1 | Target of Myb1 membrane trafficking protein | O88746 | 1.225 | 0.030 |
| Hpx | Hemopexin | Q91X72 | 1.361 | 0.011 |
| Gng4 | Guanine nucleotide-binding protein G(I)/G(S)/G(O) subunit gamma-4 | P50153 | 1.205 | 0.033 |
| Ftl1 | Ferritin light chain 1 | P29391 | 2.362 | 0.002 |
| Slc4a1 | Band 3 anion transport protein | P04919 | 1.543 | 0.005 |
| Itpr3 | Inositol 1,4,5-trisphosphate receptor type 3 | P70227 | 1.230 | 0.006 |
| Ahsg | Alpha-2-HS-glycoprotein | P29699 | 1.249 | 0.035 |
| Fgg | Fibrinogen gamma chain | Q8VCM7 | 1.387 | 0.041 |
| Slc6a6 | Sodium- and chloride-dependent taurine transporter | O35316 | 1.211 | 0.031 |
| Gigyf2 | GRB10-interacting GYF protein 2 | Q6Y7W8 | 1.350 | 0.049 |
| Gc | Vitamin D-binding protein | P21614 | 1.224 | 0.008 |
| Tnfaip8 | Tumor necrosis factor alpha-induced protein 8 | Q921Z5 | 1.204 | 0.005 |
| Zmym3 | Zinc finger MYM-type protein 3 | Q9JLM4 | 1.240 | 0.042 |
| Epb42 | Protein 4.2 | P49222 | 1.419 | 0.031 |
| Apoc1 | Apolipoprotein C-I | P34928 | 1.355 | 0.004 |

Table S22 The differentially expressed proteins in Ovx+h vs. Ovx+l mice

| Protein hydrolysates | Full name of protein | Item | Ovx+h vs. Ovx+l | |
| --- | --- | --- | --- | --- |
|  |  |  | Ratio | P |
| Mag | Myelin-associated glycoprotein | P20917 | 0.718 | 0.019 |
| Cspg5 | Chondroitin sulfate proteoglycan 5 | Q71M36 | 0.559 | 0.011 |
| Chp1 | Calcineurin B homologous protein 1 | P61022 | 0.785 | 0.016 |
| Mtmr6 | Myotubularin-related protein 6 | Q8VE11 | 0.825 | 0.013 |
| Gcc2 | GRIP and coiled-coil domain-containing protein 2 | Q8CHG3 | 0.832 | 0.001 |
| Als2 | Alsin | Q920R0 | 0.833 | 0.000 |
| Myh6 | Myosin-6 | Q02566 | 0.598 | 0.003 |
| Map3k5 | Mitogen-activated protein kinase kinase kinase 5 | O35099 | 0.783 | 0.012 |
| Lrrc8d | Volume-regulated anion channel subunit LRRC8D | Q8BGR2 | 0.786 | 0.037 |
| Tbl1x | F-box-like/WD repeat-containing protein TBL1X | Q9QXE7 | 0.797 | 0.021 |
| Sdf2l1 | Stromal cell-derived factor 2-like protein 1 | Q9ESP1 | 0.726 | 0.047 |
| Mrps28 | 28S ribosomal protein S28, mitochondrial | Q9CY16 | 0.780 | 0.037 |
| Tph2 | Tryptophan 5-hydroxylase 2 | Q8CGV2 | 0.831 | 0.046 |
| Lias | Lipoyl synthase, mitochondrial | Q99M04 | 0.782 | 0.038 |
| Tcf25 | Transcription factor 25 | Q8R3L2 | 0.803 | 0.042 |
| Nrip2 | Nuclear receptor-interacting protein 2 | Q9JHR9 | 0.747 | 0.017 |
| Ncbp2 | Nuclear cap-binding protein subunit 2 | Q9CQ49 | 0.816 | 0.014 |
| Cmc1 | COX assembly mitochondrial protein homolog | Q9CPZ8 | 0.803 | 0.010 |
| Nanp | N-acylneuraminate-9-phosphatase | Q9CPT3 | 0.767 | 0.001 |
| Zc3h18 | Zinc finger CCCH domain-containing protein 18 | Q0P678 | 0.664 | 0.009 |
| Mrtfa | Myocardin-related transcription factor A | Q8K4J6 | 0.815 | 0.004 |
| Aifm2 | Ferroptosis suppressor protein 1 | Q8BUE4 | 0.802 | 0.009 |
| Mcoln1 | Mucolipin-1 | Q99J21 | 0.782 | 0.018 |
| Kctd5 | BTB/POZ domain-containing protein KCTD5 | Q8VC57 | 0.791 | 0.015 |
| Pja1 | E3 ubiquitin-protein ligase Praja-1 | O55176 | 0.830 | 0.016 |
| Stk38 | Serine/threonine-protein kinase 38 | Q91VJ4 | 0.811 | 0.029 |
| Ftl1 | Ferritin light chain 1 | P29391 | 1.811 | 0.002 |
| Slc7a5 | Large neutral amino acids transporter small subunit 1 | Q9Z127 | 1.766 | 0.021 |
| Ldhc | L-lactate dehydrogenase C chain | P00342 | 1.391 | 0.000 |
| Timm8b | Mitochondrial import inner membrane translocase subunit Tim8 B | P62077 | 1.235 | 0.029 |
| Ppp1r12b | Protein phosphatase 1 regulatory subunit 12B | Q8BG95 | 1.229 | 0.021 |
| Nars2 | Probable asparagine--tRNA ligase, mitochondrial | Q8BGV0 | 1.251 | 0.009 |
| Thoc1 | THO complex subunit 1 | Q8R3N6 | 1.249 | 0.016 |
| Slc24a4 | Sodium/potassium/calcium exchanger 4 | Q8CGQ8 | 1.228 | 0.012 |
| Tor1aip1 | Torsin-1A-interacting protein 1 | Q921T2 | 1.255 | 0.001 |
| Npl | N-acetylneuraminate lyase | Q9DCJ9 | 1.210 | 0.037 |
| Ric3 | Protein RIC-3 | Q8BPM6 | 1.209 | 0.042 |
| Snrnp27 | U4/U6.U5 small nuclear ribonucleoprotein 27 kDa protein | Q8K194 | 1.214 | 0.009 |
| Nelfa | Negative elongation factor A | Q8BG30 | 1.393 | 0.002 |
| Oxct2a | Succinyl-CoA:3-ketoacid coenzyme A transferase 2A, mitochondrial | Q9JJN4 | 3.364 | 0.000 |
| Snx19 | Sorting nexin-19 | Q6P4T1 | 1.292 | 0.008 |
